# Determinants of intentions to prevent triatomine infestation based on the health belief model: An application in rural southern Ecuador

**DOI:** 10.1371/journal.pntd.0007987

**Published:** 2020-01-30

**Authors:** Benjamin R. Bates, Anita G. Villacís, Angela Mendez-Trivino, Luis E. Mendoza, Mario J. Grijalva

**Affiliations:** 1 School of Communication Studies, Ohio University, Athens, OH, United States of America; 2 Infectious and Tropical Disease Institute, Biomedical Sciences Department, Heritage College of Osteopathic Medicine, Ohio University, Athens, OH, United States of America; 3 Center for International Studies, Ohio University, Athens, OH, United States of America; 4 Centro de Investigación para la Salud en América Latina, Escuela de Ciencias Biológicas, Facultad de Ciencias Exactas y Naturales, Pontificia Universidad Católica del Ecuador, Quito, Ecuador; National Center for Atmospheric Research, UNITED STATES

## Abstract

**Introduction:**

Control of triatomine infestation is a key strategy for the prevention of Chagas disease (CD). To promote this strategy, it is important to know which antecedents to behavioral change are the best to emphasize when promoting prevention.

**Objective:**

The aim of this study was to determine predictors for intention to prevent home infestation based on the Health Belief Model (HBM), a commonly used health intervention planning theory.

**Materials & methods:**

A cross-sectional study was conducted with 112 heads of household in six communities with endemic and high rates of triatomine infestation in Loja province, Ecuador. The data was collected by a questionnaire including perceived severity, susceptibility, benefits to action, barriers to action, and self-efficacy. These data were also used to predict actual infestation of homes.

**Results:**

Community members reported strong intentions to prevent home infestation. HBM constructs predicted about 14% of the observed variance in intentions. Perceived susceptibility and severity did not predict behavioral intention well; perceived barriers to small-scale action that reduce likelihood of infestation and self-efficacy in participating in surveillance systems did. Self-efficacy and perception of barriers were equally powerful predictors. The HBM constructs, however, did not predict well actual infestation.

**Conclusion:**

The findings supported the HBM as a way to predict intentions to prevent infestation of the home by triatomine bugs. The findings highlight that messages emphasizing self-efficacy in participating in surveillance systems and overcoming barriers to small-scale action that reduce likelihood of infestation, rather than a focus on risk, should be central messages when designing and implementing educational interventions for CD. The gap between behavioral intention and actual infestation reveals the need to assess home practices and their actual efficacy to fully enact and apply the HBM.

## Author summary

One risk factor for Chagas disease is whether its insect vector is present in a person’s home. Although excluding these vectors–triatomine bugs–is important, we will need to know what predicts a person’s intention to exclude bugs from their homes and what kinds of messages we should use to promote intentions to exclude bugs. This study applied the Health Belief Model to identify predictors of people’s intentions. We surveyed 112 heads of household in five communities in Loja province, Ecuador, to examine five predictors of people’s intentions. We looked at perceived severity (how bad people think bugs bits are), susceptibility (how likely they think a bite is), benefits to action, barriers to action, and self-efficacy (the feeling that they can act). Community members reported strong intentions to prevent bugs from entering homes. Perceived susceptibility and severity predict behavioral intention poorly; perceived barriers and self-efficacy are better predictors. These intentions, however, do not directly lead to lower rates of infestation. Based on these findings, if we want to persuade people to try to prevent infestation of the home, we should send messages about self-efficacy in participating in surveillance systems and overcoming barriers to small-scale action that reduce likelihood of infestation. A focus on risk should not be the central message when designing and implementing educational interventions. In addition to promoting better messages, we must also attend to people’s actual behaviors and the actual efficacy of these behaviors if we want the fullest enactment of the Health Belief Model in promoting effective change.

## Introduction

Chagas disease (CD) is a neglected tropical disease that affects approximately 8 million people worldwide, but that is found mostly in Latin America [1]. The disease is caused by the *Trypanosoma cruzi* parasite [2]. *T*. *cruzi* is transmitted by triatomine insects (Reduviidae: Triatominae). Triatomine insects are nocturnal blood-sucking insects whose range extends from the southern United States to northern Chile and Argentina [1]. In Ecuador, about 200,000 people are infected with *T*. *cruzi*, and at least 3.8 million are at risk of infection according to the current estimates [3]. Sixteen species of Triatominae, known in Ecuador as *chinchorros*, have been reported in Ecuador, distributed in twenty of the twenty-four provinces [4].

Triatomine bugs inhabit poorly constructed homes. The bugs live in the cracks of the walls, ceilings, and floors of abode houses, and when these cracks are present they allow triatomines to invade and colonize people’s homes [5–8]. The bugs emerge at night to feed. *T*. *cruzi* infection occurs when the triatomine bug defecates during or immediately after feeding. The person bit then scratches the triatomine feces into the bite or into the mucous membranes of the eyes or mouth, thus introducing the *T*. *cruzi* parasite into the body [1]. The acute symptoms of CD are largely mild and nonspecific but, over time, there could be digestive tract alterations such as megacolon and megaesophagus, and cardiac disorders, particularly megacardia and heart failure. These pathological alterations decrease the quality of life and, ultimately, lead to death in many cases [9]. Due to limitations of access to diagnosis, [10] treatment is seldom administered.

Recent research has concluded that influencing health behaviors and improving housing infrastructure are potential mechanisms to prevent and control CD [11]. Furthermore, the qualitative results found by Patterson et al. indicate that the connection between the presence of triatomines in the home, their feeding and transmission of the *T*. *cruzi* parasite, and later development of CD is not well understood by community members [11]. Therefore, understanding perceptions that go beyond the immediate risk of being exposed to the vector (the triatomine) may allow us to develop messages based on these perceptions. Following the Patterson et al.’s arguments regarding the importance of perceptions of antecedents to preventive action, the present study uses the Health Belief Model (HBM) to examine five predictors of people’s intentions to exclude bugs from their homes in order to inform what kinds of messages should be used to promote intentions to exclude bugs.

The World Health Organization recognizes that vector control is essential to stopping CD [1]. The main implementation of vector control has been the regular spraying of homes with residual insecticides to make homes unwelcome to triatomine species [12]. These efforts are costly and they are largely reactive to the presence of triatomines [13]. To complicate matters, several endemic species of triatomines have sylvatic (wild) populations, and readily re-invade homes once the residual effect of the insecticide subsides [4, 13]. Insecticide spraying based control interventions do not address root causes of infestation. These root causes are in part behavioral. Practices in the communities make homes conducive to triatomine infestation, including the accumulation of materials in the home, such as extra clothing or building supplies, which creates hiding places for triatomines during the day, and the accumulation of foodstuffs that attract rodents and small animals that can act as hosts for triatomines [14–16]. Animal husbandry practices, such as keeping chicken nests against the walls of the home, keeping Guinea pigs in the house or allowing pigs and goats to move from the wild to the peridomicile to the home [15], attract triatomines. Precarious house construction allows invading triatomines to hide in cracks in the walls and dark, cluttered spaces prevent their detection by dwellers. Altogether, these conditions allow triatomines to establish colonies in and around the house, perpetuating CD transmission.

Although behavior change appears to be essential to excluding triatomine bugs from individual homes, little is known about why some people engage in preventive behaviors and others do not. This research, therefore, seeks to find factors most predictive of behavioral change in the context of excluding bugs. Drawing on the HBM, and particularly Patterson et al.’s [11] application of this model in southern Ecuador, we seek to understand which behavioral antecedents are most predictive of intention to safeguard one’s home from bugs and, thereby, serve as effective pathways for persuading heads of households to alter their behaviors. After reviewing the HBM, we use a survey-based approach to determine which, if any, of the antecedents to behavioral change are most predictive of intentions to exclude bugs. Based on our findings, we offer suggestions for interventions that seek to effectively communicate the risk of, and viable prevention measures for, the control of triatomine vectors.

### The health belief model

The HBM provides an explanatory framework to understand why some individuals in these communities intend to prevent triatomine infestation and others do not. Relevant to the specific context, the HBM has been used successfully across Latin America and, specifically, in Ecuador [11, 17–19], and its constructs are translatable and applicable to Spanish-speaking populations [20, 21]. The HBM argues that there are five antecedents to behavior change [22, 23]. “Perceived susceptibility” assesses how likely a person believes that she is to be exposed to a health condition or its outcomes. “Perceived severity” assesses how strong the negative impact of that exposure is seen to be. When perceived susceptibility is combined with perceived severity, higher susceptibility to highly severe outcomes should promote intention to change behavior, but when either susceptibility or severity is perceived to be low, intentions to change behavior are less likely. The third antecedent is the “perception of barriers”, or obstacles to performing a recommended behavior. Barriers may derive from perception of time, reputational, or financial cost, among others. Barriers are weighed against the fourth component: “perceived benefits”. Perceived benefits are the positive outcomes that are anticipated to emerge from adopting the recommended behavior. If the person believes the benefits outweigh the barriers, they are likely to intend to adopt a recommended action. Finally, “self-efficacy”, or one’s own perception that they can perform the behavior, can influence the adopting of the recommended behavior. A person who believes that they have the knowledge and skills to perform the recommended behavior is more likely to intend to do so than a person who does not believe they possess that knowledge or skill set.

Understanding these first five antecedents can inspire messages (called “cues to action” in the HBM) to promote behavior changes. Moreover, assessing which antecedents, if any, are most influential can help to refine those messages and make them more likely to succeed. Thus, in the context of excluding triatomines, we pose the following research question:

RQ: Which HBM variables, if any, predict behavioral intention to exclude triatomines from the home, and which, if any, is the best predictor?

## Methods

### Ethics statement

All procedures and protocols for this study were approved by the Ohio University Institutional Review Board (18-D-80) and the Research Ethics Committee at the Pontifical Catholic University of Ecuador (CEISH 2018-35-EO). All participants were adults. Consent documents, in Spanish, were read to participants and were provided to the participants who wished to read them. Participants indicated their consent by either signing the form or stamping it with their thumbprint.

### Study site

**Fig 1:**
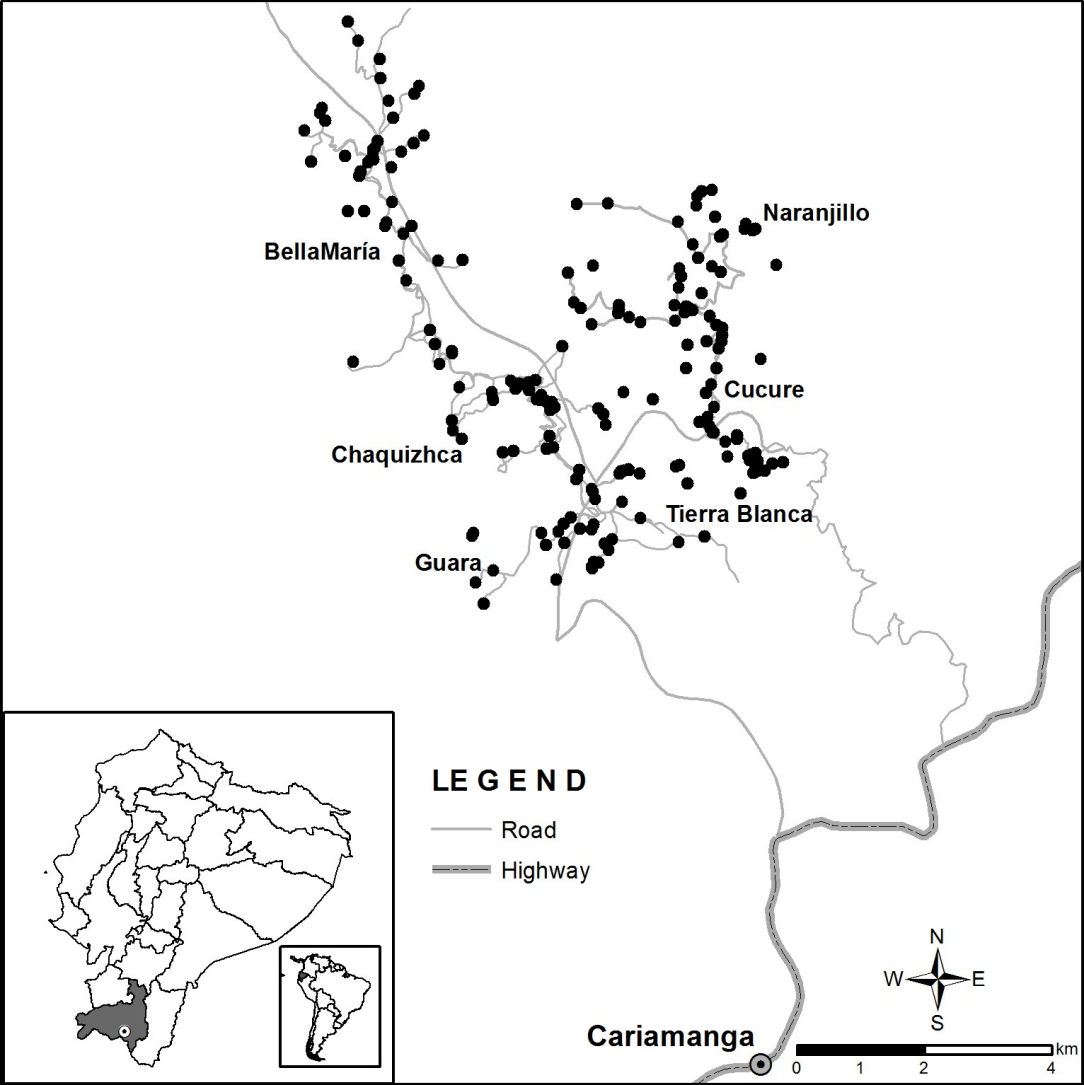
Map of the Communities in relation to nearest city of 25,000 residents.

This study was conducted in six rural communities in Loja province, Ecuador (Fig 1). Loja province has a high rate of triatomine infestation and, thereby, is likely to experience high rates of CD (overall infection rate, 8.8%) [15]. Three of the communities (Guara, Chaquizhca and Bellamaria) have been visited by our project team since 2010 as pilot communities for interventions to prevent home infestation. The other communities have been more recently involved, either since 2014 (Naranjillo) or newly visited in 2018 (Cucure and Tierra Blanca). All of these communities are located in Ecuador’s southern highlands: mountainous terrain with limited road, water and sanitation infrastructure. The deep dendritic ridges and winding, unpaved roads and pathways make travel and transportation difficult; community members often must walk one or two hours to visit one another or to get to the main road to find transportation to the nearest town of more than 5,000 people. Most of the homes in these communities have structural, behavioral, and/or peridomiciliary conditions that place them at greater risk of triatomine infestation. Structurally, most of the homes in these communities are adobe constructions, with dirt floors, walls raised on found stone foundations, ceilings made of bamboo, and roofs made of loosely fitted tiles [24]. Many of the families enact behaviors that attract hosts for triatomines, such as cohabitation of these homes with domestic animals and the accumulation of grain and produce in the home (which attracts mice, rats, and squirrels) [25]. And, in the peridomicile, the presence of pigs, goats, and other livestock is common [15]. Each of these factors raises risk of home infestation by triatomines.

### Participants

The project team, with the assistance of the Ministry of Public Health of Ecuador, deployed the questionnaire during visits to homes in summer 2018. All homes in these six communities were visited by the Ministry and members of the research team. To obtain data for other projects, each home received an entomological inspection to determine the presence or absence of triatomines, as well as an assessment of the home’s construction and peridomicile to assess factors that make individual houses more or less susceptible to infestation. While the home’s features were inspected, for the present study, the head of household was asked the 16 questions about their beliefs and perceptions about exposure to triatomine bites (questionnaire described below). Although the ultimate goal is to prevent the transmission of CD, previous interview research indicates that the connection between the presence of triatomines in the home, their feeding and transmission of the *T cruzi* parasite, and later development of CD is not well understood in Chaquizhca, Bellamaria, and Guara [11]. Understanding of these connection in Nanjillo, Tierra Blanca, and Cucure is unknown. Understanding perceptions that go beyond the immediate risk of being exposed to the vector (the triatomine) may allow us to develop messages based on these perceptions.

Approximately half of the households in in which people were resident (i.e., excluding abandoned and unoccupied homes and those that refused to participate in entomological searches) agreed to participate in our questionnaire (see Table 1). Two hundred and two homes were searched for bugs and one hundred twelve heads of household agreed to participate in the questionnaire portion of the study. A substantial number of homes were infested by one or more species of triatomine bugs, ranging from a low of 14.3% in Bellamaria [4 of 28 homes searched] to a high of 31.3% in Naranjillo [21 of 67 homes searched] (see Table 1). All triatomines discovered in these searches were either *Rhodnius ecuadoriensis*, a domiciliary colonizing species, or *Panstrongylus chinai*, a domiciliary non-colonizing species. The presence of these bugs is similar to that found in a previous comprehensive survey of these communities, which are found only *R*. *ecuadoriensis* and *P*. *chinai* infesting homes in these communities [15]. Communities were well represented in the questionnaire: Bellamaria (n = 22; 78.6% of occupied homes), Chaquizhca (n = 24; 61.5%), Guara (n = 25; 78.1%), Naranjillo (n = 31; 46.3%), Tierra Blanca (n = 4; 20.0%), Cucure (n = 6; 37.5%). Generally, there was more participation from communities that have been involved in the project for a longer period of time, Fisher’s exact test revealed that this difference was statistically significant (50.3% (71/141) of households participating in the questionnaire in long-term communities, compared to 27.5% (41/159) in short-term communities (p < .01) and, therefore, long-term participation versus shorter-term participation of the community was included as a variable in the later regression analysis. Infestation rates were lower in communities where the project has been involved for a longer period of time, however differences in infestation rates across communities were not significant (23.2% (23/99) of homes searched in longer term communities were infested, compared to 31.1% (32/103 in shorter-term communities, p = .27). Of these heads of household, 56 were men and 50 were women. Household sizes ranged from single person homes to those with ten residents (Median = 3.0, IQR = 2.0–5.0). All households were rural, practiced subsistence agriculture, and had household incomes below Ecuador’s poverty line.

**Table 1:** Total number of houses, houses searched for triatomines, households participating in survey, and infestation rates.

Community	Number of total houses in community	Number of houses searched for triatomines	Number of households participating in in survey	Infestation rate (%)
Guara	52	32	25	25
Chaquizhca	51	39	24	28.2
Bellamaría	38	28	22	14.3
Naranjillo	109	67	31	31.34
Cucuré	26	16	6	31.25
Tierra Blanca	24	20	4	30
TOTAL	300	202	112	27.22%

Note: Number of houses in community includes abandoned and uninhabited houses

### Questionnaire development

The present data was collected in the context of a nearly 20-year long research and service-learning collaboration between Ohio University’s Infectious and Tropical Disease Institute (ITDI),the Pontificia Universidad del Ecuador’s “Centro de Investigación para la Salud en America Latina” (CISeAL), and their partner communities in Loja, Ecuador. The center of this large participatory research intervention is the Healthy Living Initiative (HLI), an ongoing, multidisciplinary research initiative that supports sustainable community education, socioeconomic development, and housing (re)construction in rural communities as its main tools for the control of CD (see, for example, [11, 15, 16, 24, 26–28]. In this ongoing engagement with communities in Loja, our project team has adopted the principle that constructs of health and healthy living are best articulated by members of the community so that culture-centered, community-appropriate interventions can be designed [27]. Through continuous engagement with youth [28] and adults [16] in these communities, we have been able to understand better their perceptions and operationalizations of concepts health, risk, prevention practices, barriers to practice, and what insect exclusion from homes mean [26, 27]. Allowing communities to define these terms, rather than having a researcher impose the researcher’s vocabulary, allows the community articulate its lexicon for persuasion, its level of knowledge, and its framework for salient message design [29–31].

Most specific as a precursor to the present data set was the work performed by Patterson et a. [11]. Patterson et al.’s data, originally collected in 2014, used a focus group approach to identify community perceptions of CD and the practices community members understood as important opportunities for or barriers to insect exclusion. Patterson et al engaged three of the communities involved in the present study: Chaquizhca, Guara, and Bellamaria. During the focus groups, Paterson et al argued that the community developed a common vocabulary around what it would mean to exclude triatomine bugs from their homes and, by understanding and employing this vocabulary, the project team could use terms and expressions used by community members. In this focus group study, community members viewed educational videos, articulated practices that were part of excluding insects from their homes, and indicate behaviors that were easy and those that were difficult to adopt. Patterson et al then coded community contributions into HBM constructs of threat (a combination of severity and susceptibility), perceived benefits and barriers, and self-efficacy.

**Fig 2:**
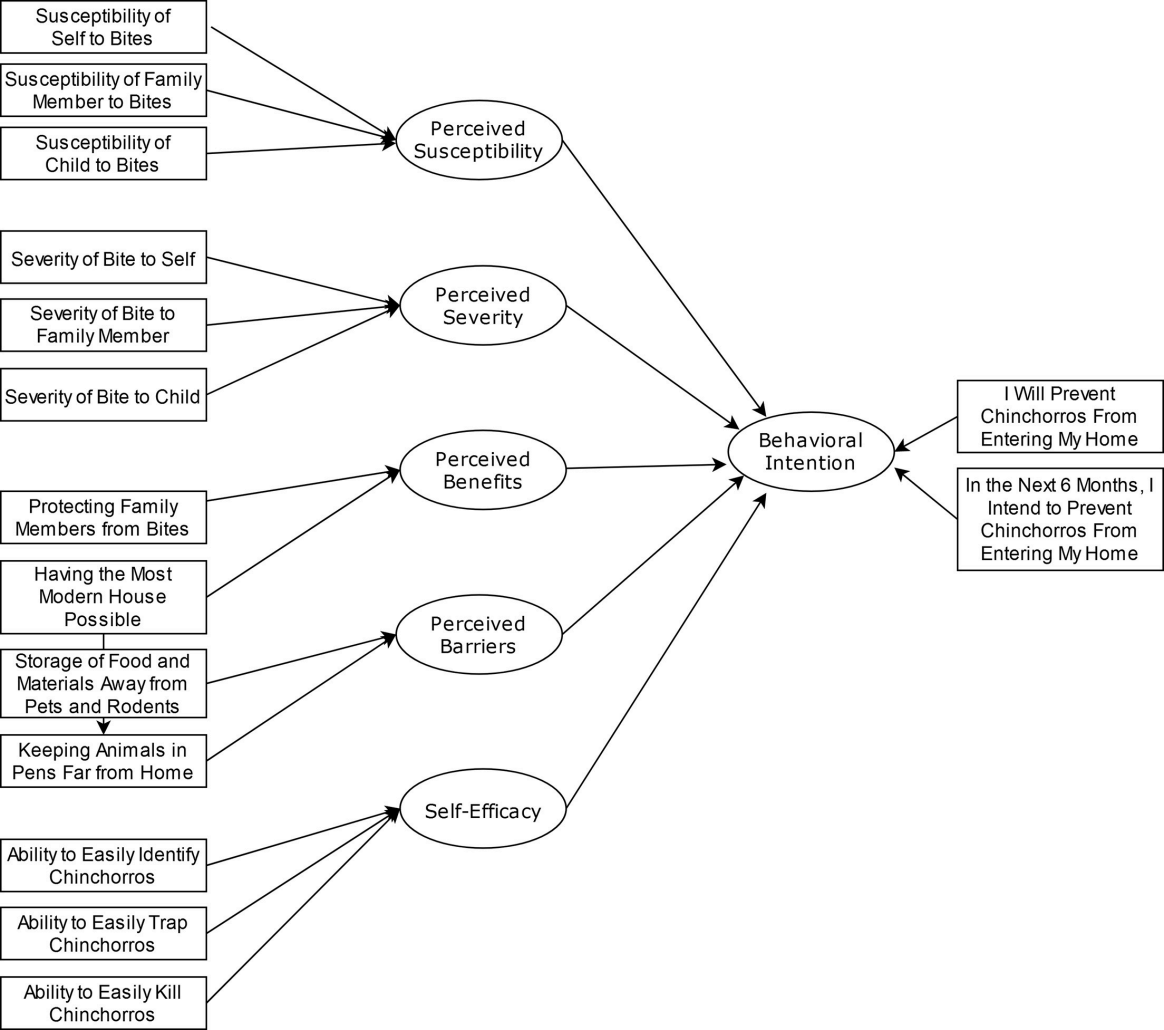
Relationships among HBM concepts and HBM questionnaire items.

Following standard questionnaire development practices [32], these qualitative observations were transformed into a set of measures that reflect the local understanding of the constructs of interest [29–31]. Regarding threat, Patterson et al found that the three salient targets were self, family, and children, and that the community believed there could be different harms (severity) and levels of risk (susceptibility) for these three targets. These three targets then informed the three severity items and the three susceptibility items. In reference to barriers, Patterson et al’s participants identified food storage and animal containment practices as significant barriers to triatomine exclusion. These two difficult practices were used to represent the construct of barriers in the questionnaire. For benefits, community members identified the two main benefits of triatomine exclusion as protecting the family’s health and having a modern home. These two benefits were used to represent the construct of benefits. Because community members had been previously taught how to identify triatomines, how to trap them (and provided with rubber gloves, a plastic jar, and instructions on how to deliver suspected triatomines to the project team’s surveillance system), and how to properly kill them (i.e., to not squish bugs with their bare hands), these were used to inform the construct self-efficacy [16]. Finally, two items reflecting the community’s overall conceptualization of intention were created. All items were measured using a 1 (completely disagree) to 4 (completely agree) scale. The Appendix contains the full questionnaire wording. The conceptual fit among the questionnaire items and the HBM constructs is displayed in Fig 2. This questionnaire was deployed in summer 2018 to collect the present data.

### Measures

#### Perceived susceptibility

Although triatomines are common in both the domestic and sylvatic environments of Loja province, not all members of the community believe that the bugs could enter their homes and bite them. Therefore, perceived susceptibility was measured using three items. These items referred to three kinds of persons identified in Patterson et al.’s study who could be bitten by a triatomine–self, family, and child–and assessed the participant’s perception of likelihood that each could be bitten by a triatomine in the future.

#### Perceived severity

Even if a person sees a triatomine in their home and is bitten, they may not believe that being bitten is a serious problem. Although the bite itself does not transmit CD, the total number of feedings by triatomines increases the probability that a human host will become infected. Therefore, crafting an association between bites and CD can serve as a useful shorthand for why the bugs and their feeding is dangerous [33]. Perceived severity was measured using three items. These items referred to the same persons who could be bitten and assessed the participant’s perception of the severity of being bitten.

#### Perceived benefits

If a person anticipates positive outcomes from adopting the recommended measure, in this case triatomine exclusion from the home, she may be more likely to adopt the action. To measure the perceived benefits of excluding triatomines, two items were used. These items referred to two primary benefits named in Patterson et al.’s study: protecting family health and having a modern home.

#### Perceived barriers

If a person believes that there are significant factors that prevent her from adopting a preventive measure, she may be less likely to adopt the action. To measure the perceived barriers to triatomine exclusion, the same scale was used, but the answers were reversed for analysis as the items asked about abilities to perform recommended actions rather than disabilities. These items used the two barriers named most often in Patterson et al.’s study: difficulties in storing foodstuffs in the home where domestic animals and rodents cannot get to them and in keeping domestic animals stored in pens away from the home.

#### Self-efficacy

Self-efficacy refers to a person’s beliefs about their ability to accomplish a particular task or tasks related to the preventive measure. To measure the sense of self-efficacy, participants were asked about their ability to perform three actions related to triatomine exclusion identified in Patterson et al.’s study: their ability to identify, to trap, and to kill triatomines.

#### Behavioral intention

Behavioral intention, as the proximate predictor of actual behavior, was employed as the dependent variable. Although behavioral intention is not the same as behavior, previous meta-analyses indicate behavioral intention is an excellent predictor of behavior in both prospective and retrospective studies [34–36]. In addition, previous research indicates that predictor variables do not directly predict behavior; rather, predictor variables predict behavioral intention, which, in turn, predicts behavior [37, 38]. Two items were used to measure intention to exclude triatomines from the home. One was immediate intention (I will prevent triatomines from entering my home) and one was in the future (in the next six months, I intend to keep triatomines from entering my home).

### Analytic procedures

In analysis, we first assessed measures for reliability. Following generally accepted guidelines for behavioral research in public health [39–41], three-item measures were evaluated using Cronbach’s alpha (α) and two-item measures were evaluated using Spearman-Brown rho (ρ). Although there are few firm reliability cut-offs [42], if a measure displayed inter-item correlation below .40 for either measure of consistency the measure was excluded from further analyses. We then examined the correlations among measures and assessed possible multicolinearity. Potential multicolinearity among explanatory variables was evaluated through the variance inflation factor (VIF). A visual examination of the P-P plot was employed to test the whether the relationship between the independent and dependent variables be linear. Normality of the data was checked with the Kolmogorov-Smirnov test. Autocorrelation was tested with the Durbin-Watson test (d). An examination of the scatterplot was used to assess whether the data as a whole are homoscedastic.

Finally, a multiple linear regression analysis was employed with behavioral intention set as an ordinal outcome. In the first block, we included gender, household size, and community of residence as demographic variables. In the second block, we included HBM measures (described below) if the measures met standard assumptions of reliability. Because there are a small number of predictors and the research question asks which predictor is the strongest, we employed the enter method, sometimes called “forced entry,” to assess each predictor. The enter method is appropriate when there are theoretical reasons to expect that every variable influences the outcome and when there are a small number (<20) variables under consideration. It also avoids the potential suppressive and inflationary effects, respectively, of backward stepwise and forward stepwise regression [43, 44].

## Results

### Reliability

The candidate scales were first examined for reliability. The measures of perceived susceptibility (α = .90) and perceived severity (α = .91) we highly reliable. The measures of perceived barriers (ρ = .67) and self-efficacy (α = .65), were moderately reliable. The measure of behavioral intentions was sufficiently reliable (ρ = .57). These variables were all retained for further analysis. The two items measuring perceived benefits failed to form a reliable measure (ρ = .32). Although single-item measures are rarely adequate to capture a construct, the face validity of the benefit “protecting family health” indicates it may function differently than the benefit “having a modern home.” If this item correlated well with both severity and susceptibility, it could be included as a single-item measure. The single item was correlated with susceptibility (r = .31, p < .05) but not severity (r = .11). Given that, in this construction, the named benefit does not meet the theoretical explanation for why this benefit would be the inverse of total threat, we did not retain it for the regression model. We, therefore, did not include perceived benefit in the further analyses.

### Means and correlation

Final variables were computed by calculating the mean of all answers to the individual items that compose the scale, resulting in a scale score between 1 and 4. Generally, participants expressed moderate agreement that they and their families were susceptible to triatomine bites (μ = 2.98, s.d. = 1.14) and agreed strongly that triatomine bites would have severe consequences (μ = 3.60, s.d. = 0.79). They saw significant barriers to infestation prevention (μ = 3.14, s.d. = 1.07), but also had a strong sense of self-efficacy in engaging in prevention behaviors (μ = 3.38, s.d. = 0.83). Finally, they expressed strong intentions to prevent infestation of their homes (μ = 3.22, s.d. = 0.92).

As indicated in Table 2, there were some significant correlations among some explanatory variables. Specifically, there was a moderate association between severity and susceptibility, between perceived berries and behavioral intention, and between self-efficacy and behavioral intention. The VIF was < 2 for all explanatory variables, well below the standard conservative VIF cut-off of VIF = 5 [45]. In addition, each individual correlation is well below conservative cut-off points if inter-measure correlation of .50 [46]. Together, these indicators show that the significant correlation found between some pairs of variables would not cause serious multicolinearity.

**Table 2:** Means, medians, ranges, standard deviations, and correlations of HBM constructs.

Variable	μ	M	Range	IQR	SD	1	2	3	4
1. Severity	3.59	4.00	1.0–4.0	3.00–4.00	.79	--			
2. Susceptibility	3.00	3.33	1.0–4.0	2.33–4.00	1.12	.28**	--		
3. Barriers	3.19	3.50	1.0–4.0	2.50–4.00	1.04	.07	.04	--	
4. Efficacy	3.40	4.00	1.0–4.0	3.00–4.00	.80	.13	.04	.06	--
5. Intention	3.22	3.50	1.0–4.0	2.50–4.00	.92	-.00	.07	.24*	.26**

Note: μ, M, SD, and IQR are used to represent mean, median, standard deviation, and inter-quartile range, respectively.

* indicates *p* < .05.

** indicates *p* < .01.

We also assessed whether there were differences between communities in their responses to the questionnaire. A Kruskal-Wallis H test indicated that there was not a statistically significant difference among communities in their scores by site (Susceptibility, χ2(3) = 1.37, p = .71; Severity, χ2(3) = 3.98, p = .26; Efficacy, χ2(3) = .07, p = 1.00; Barriers, χ2(3) = .65. p = .88; Intention, χ2(3) = 3.59, p = .31) [two communities had insufficient numbers to allow the post hoc Kruskal-Wallis H test]). We therefore chose not to use a full multi-level modeling approach in the regression analysis.

### Regression

Before reporting the results of the regression, we assessed whether the assumptions for this test were met. A visual examination of the P-P plot indicated that points followed the normality line without drastic deviation, thus meeting the assumption that the relationship between the independent and dependent variables be linear. Second, normality was checked with the Kolmogorov-Smirnov test. Skewness of z > |2.8| or kurtosis > |3.00| indicates data are not normally distributed [47, 48]. All variables were within acceptable ranges and did not differ from a normal distribution. Third, linear regression assumes that there is little or no multicollinearity in the data; as indicated above, the VIF < 2 for all explanatory variables, indicates there is little multi-collinearity. We tested the linear regression model for autocorrelation with the Durbin-Watson test (*d*). Values of 1.5 < *d* < 2.5 show that there is no autocorrelation in the data. In the model, *d* = 1.57, indicating no autocorrelation in the data. Finally, an examination of the scatterplot revealed no obvious pattern to the data, with approximately equal numbers of points distributed above and below the X axis and to the left and right of the Y axis. The data as a whole is homoscedastic. Thus, the assumptions for regression analysis were met.

The results of the regression indicated that the HBM predictors explained about 12% of the variance in behavioral intentions (R^2^ = .12, F (8,81) = 2.70, p = .02) (see Table 3). Neither gender (β = -.03, p = .86) nor household size (β = .13, p = .22) predicted behavioral intention. In the initial solution, community of residence (shorter-term vs. longer-term partner communities) was significant (β = .18, p = .04), indicating that people living in communities with a longer-term relationship with the overall project were more likely to intend to exclude triatomines from the home, this difference was no longer significant once HBM variables were entered (β = .17, p = .10). Within the HBM variables, an individual’s sense of barriers (β = .21, p = .02) and of efficacy (β = .23, p = .05) predicted behavioral intention. That is, a belief that barriers could be overcome and that the individual had the ability to enact recommended behaviors predicted intention to exclude triatomine bugs from the home. However, the participants’ sense of susceptibility to triatomine bites (β = .10, p = .38) or of the severity of bites (β = -.08, p = .54) did not predict behavioral intentions.

**Table 3:** Regression results using intention to keep home free of triatomines as the criterion.

	Model 1			Model 2		
Predictor	*B*	β	p	*B*	β	p
1^st^ Block: Demographics						
Sex	.11	.06	.57	.03	.02	.86
Size of household	.08	.17	.11	.06	.13	.22
Community of Residence	.18	.22	.04	.14	.17	.10
2^nd^ Block: HBM						
Severity				-.08	-.07	.54
Susceptibility				.08	.10	.38
Barriers				.21	.25	.02
Efficacy				.23	.20	.05
ΔR^2^	.07, p = .10			.12, p = .02		
Total R^2^ adjusted				.12, p = .02		

Note: HBM = Health Belief Model.

Finally, a binomial logistic regression was performed to ascertain the effects of community of residence (shorter-term vs. longer-term partner communities), perception of barriers to action related to preventing triatomines’ entry into homes, perceptions of self-efficacy in participating in surveillance and control systems, and behavioral intention on the likelihood that an individual participant’s home would be infested. The logistic regression model was not statistically significant, χ^2^(4) = 2.46, *p* = .65. The model explained 3.0% (Nagelkerke *R*^*2*^) of the variance in infestation yet correctly classified 66.1% of cases. This finding indicates that there is a breakdown between behavioral intention and expected outcome; actual behaviors were not measured.

## Discussion

In their tenth year summary of CD prevention research, a review period from the founding of PLoS:NTD, Dumonteil and Herrera [49] found that vector control through insecticide spraying continues to be the main means of CD preventive intervention. Although spraying seeks, and sometimes obtains, elimination of triatomines from people’s homes, even the most effective spraying campaigns may be unsustainable and, because of potential for reentry and reinfestation, ineffective in the long-term [50]. This is particularly true for areas where there are sylvatic triatomine populations that readily infest homes after the residual effect of the insecticide subsides [4, 13]. The communities where this study was conducted are located in such an area.

To address the limitations of spraying campaigns, newer interventions in Latin America have focused on training in identification of the insects and determining which homes and people are at greatest risk. The central idea is that, if members of the public understand what CD is and how it is transmitted, this knowledge will reduce infection rates [51]. In addition to community members, these educational interventions can include professional home inspectors [52] and physicians [53, 54]. Although the causal mechanism is rarely explained, researchers argue that, if individuals become more aware of the severity of CD and their susceptibility to the disease, they will be more likely to seek preventive measures [55–57]. This focus on training and home inspection is supported by the initial regression solution in this study. Longer engagement with communities, in and of itself, is strongly associated with greater intentions to prevent triatomines from entering the home. This association does not tell us, however, what about that training and inspection leads to these intentions. In the literature around training and inspection, the potential explanatory mechanisms for this connection are rarely tested.

The connection between knowledge of CD and triatomine bugs to prevention is not always a direct one. For example, Dias et al. [58] found that in Minas Gerais, Brazil, being able to identify triatomine bugs was not associated with home infestation and, further, that there was no association between knowledge of CD transmission mechanism and home infestation. Conversely, Donovan et al. [59] found in La Hicaca, Honduras that communities with high knowledge have a high desire for prevention and testing, but did not assess an association with infestation rates or prevention practices. Because of this lack of simple causal relationship, there is a growing interest in other aspects of communication intervention, including community norms campaigns [60] and stigma reduction [61].

The present study addresses both the lack of a causal mechanism between knowledge and prevention intentions and tests whether severity and susceptibility are the best areas of focus. Individuals in these six communities understood, generally, that they were susceptible to triatomine bites and that being bitten could lead to severe consequences. This knowledge, however, did not predict whether individuals would intend to prevent bugs from entering their home. Instead, it was when individuals believed that they could overcome barriers to action and that they had the ability to act that they intended to prevent home infestation. To answer the research question formally, the HBM variables that predict behavioral intention to exclude triatomines from the home are the individual’s sense of barriers to acting to prevent bugs from entering their home and of self-efficacy in identifying and killing bugs that had entered, and these two variables are approximately equal in strength. However, the HBM variables and behavioral intention did not predict the expected outcomes of infestation or no infestation. Although actual behaviors were not measured, it is likely that there is either a gap between intention and ability to enact the recommended behaviors or a gap between the enacted behavior and its actual efficacy in preventing infestation.

This pattern suggests a different mechanism for more effective persuasion and education in the community if we wish to shift behavioral intentions. Our data suggest that we should not focus on risk when communicating with members of the communities. Current emphases on the dangers of CD may not be helpful, particularly since it is a silent disease. Rather, our data suggest that it would be better to focus on reducing perceived barriers to action related to preventing triatomines’ entry into homes and encouraging self-efficacy in participating in surveillance and control systems in these communities. An examination of the educational materials employed by the project team may make these recommendations more concrete. As part of the overall intervention in 2014, each participating household received as ten-minute educational talk about triatomines and CD. They also received as booklet containing, among other features, color life-size pictures of local triatomine species (from nymph through adulthood), text- and cartoon-based explanations about the CD transmission cycle, and instructions for trapping and reporting triatomines to the local surveillance system. This set of features engages the household’s ability to identify infestations and report to authorities, thus representing an opportunity to enhance the self-efficacy of households in participating in surveillance and control systems for triatomines. A stronger and more regular focus on identification of triatomines and how to trap and report them may further enhance self-efficacy. The booklet also contained practical recommendations for preventing triatomine infestations (such as reduction of clutter in the home, relocation of domestic animals, and appropriate storage of foodstuffs and seed grain). By providing these practical recommendations, the booklet sought to reduce barriers to action by providing easy-to-implement actions that would decrease the likelihood of triatomines entering the home. By emphasizing practical recommendations, and how to implement them, the project can help community members adopt actions that reduce their risk of triatomine entry and subsequent infestation and provide alternatives to actions that may be seen as significant barriers. The findings indicate that encouraging small-scale solutions can increase intention to exclude triatomines from the home and provide pathways to adopting these actions. Thus, small-scale individual- and household-level actions can complement large-scale solutions, such as home reconstruction (which has a significant affordability barrier) or governmental spraying campaigns (which place the solution outside of the community’s control). Overall, our research suggests that, if we merely scare people about bugs and CD, we are unlikely to see attempts to act, but if we help people see how they can take effective small-scale action to eliminate bugs from their home and to see how that action could be effective we are likely to see attempts to act.

We cannot, however, be sure that intention to act will lead to the prevention of home infestation. Indeed, although the HBM predicts that behavioral intention should lead to behavior leading, in turn, to desired outcomes, there are many factors between intention and outcome that may interfere with a psychosocial level intervention based on the HBM. Because our study did not assess actual behaviors, we cannot directly assess the external validity of HBM predictors to actual behavior or the effect of these behaviors on infestation. We cannot assert whether there is a break down between intention and behavior or between behavior and actual efficacy. Future research should assess which households have reduced foodstuff and materials clutter in an attempt to reduce risk of infestation, as well as whether there is a bright line of sufficient clutter reduction to prevent likelihood of infestation. It should also assess which households keep their animals far from the house, as well as whether the distance for what constitutes “far” impacts likelihood of infestation. We also did not perform skills testing regarding actual ability to identify triatomines or actual ability to correctly capture or kill them. This means, similarly, we cannot provide a full causal pathway. There are also other behaviors that may matter but that are not measured in the present study. For example, previous research in these communities has also found that defecation behaviors, self-spraying of insecticide, and type of animal kept (pigs, goats, and sheep) affect the likelihood of infestation by *R*. *ecuadoriensis* [15]. What this study had identified, though, is a gap in the causal chain from antecedents -> health beliefs -> behavioral intentions -> behavior -> desired outcome, specifically the gap at the behavior stage. This is a significant area for future research to allow us to fully exercise the HBM.

This recommendation should be tempered by four factors. First, although our conclusion agrees with researchers in other contexts who found no association between knowledge and home infestation [58, 59], our research was limited to six communities nearby to each other. Other communities may reflect different or alternative associations than the ones in which we worked. In particular, because we have engaged in educational campaigns with three of these communities for a long period of time, we may have exhausted abilities to increase perceptions of risk in the communities. Although this is statistically unlikely in our communities, it is important to assess levels of risk and levels of efficacy for participating in the identification and surveillance systems and barriers to small-scale actions that decrease the likelihood of triatomine entry in any community before a health intervention is performed. Second, we assessed behavioral intentions to prevent triatomine bugs from entering the home, not actual behaviors. Although behavioral intention is often used as an outcome variable in HBM studies [37], and although behavioral intention is a strong predictor of actual behavior [38], there may be gaps between what an individual intends to do and what he or she actually does. Future research may wish to assess the full chain of prediction with a retrospective design that assesses presence of HBM predictor variables and the behavioral intentions found here and whether those behaviors have been performed, as well as whether those behaviors are, indeed, associated with lower rates of infestation. Specifically, future research could support the reduction of barriers pathway by assessing actual behaviors in which households have reduced foodstuff/materials clutter (or enacted different amounts of clutter reduction) or which households keep their animals far from the house (or how far away animals are kept) and associating these with both likelihood of infestation and the HBM variables. Similarly, assessing through skills testing actual ability to identify triatomines or actual ability to correctly capture or kill them and associating these skills with both likelihood of infestation and the HBM variables would better support the pathway to change through promoting of self-efficacy. The current research is probative, but cannot provide the full causal pathway. Third, although our participants comprise about half of the heads of households in these communities, our total sample is somewhat small. Larger samples may lead to more significant associations, although the p-values found for non-significant associations in the present research indicate this is unlikely. Finally, some of our measures had lower reliabilities that desired. Our reliabilities, however, are consistent with or higher than other applications of the HBM in Latin American contexts [20, 21]. Despite these limitations, our research provides some potentially powerful loci for communication and education messaging to help promote intentions to prevent triatomine bugs from entering the home.

## Supporting information

S1 File Health belief model questionnaire. (DOCX)
