# Supplementary material for: Determinants of intentions to prevent triatomine infestation based on the health belief model: An application in rural southern Ecuador
Source: PLoS Negl Trop Dis. 2020 Jan 30;14(1):e0007987. doi: 10.1371/journal.pntd.0007987 (PMC6991950; doi:10.1371/journal.pntd.0007987)
Supplement: S1 File — (DOCX) [file pntd.0007987.s001.docx]

Cuestionario del modelo creencias de la salud (HBM) sobre la exposición al triatómino responsable de la trasmisión de la enfermedad de Chagas.

Todos los ítems

Totalmente en desacuerdo-------1---

Parcialmente en desacuerdo-----2---

Parcialmente de acuerdo---------3---

Totalmente de acuerdo-----------4---

**Susceptibilidad**

1. Existe la posibilidad de que sea picado por el chinchorro en el futuro.

Totalmente en desacuerdo-------1---

Parcialmente en desacuerdo-----2---

Parcialmente de acuerdo---------3---

Totalmente de acuerdo-----------4---

2. Un miembro de mi familia puede estar expuesto a la picadura del chinchorro en el futuro.

Totalmente en desacuerdo-------1---

Parcialmente en desacuerdo-----2---

Parcialmente de acuerdo---------3---

Totalmente de acuerdo-----------4---

3. Mi hijo/hija podría ser picado por un chinchorro en el futuro.

Totalmente en desacuerdo-------1---

Parcialmente en desacuerdo-----2---

Parcialmente de acuerdo---------3---

Totalmente de acuerdo-----------4---

**Severidad**

4. Ser picado por el chichorro podría tener problemas serios de salud para mi

Totalmente en desacuerdo-------1---

Parcialmente en desacuerdo-----2---

Parcialmente de acuerdo---------3---

Totalmente de acuerdo-----------4---

5. Ser picado por el chichorro podría tener problemas serios de salud para algún miembro de mi familia.

Totalmente en desacuerdo-------1---

Parcialmente en desacuerdo-----2---

Parcialmente de acuerdo---------3---

Totalmente de acuerdo-----------4---

6. Ser picado por el chichorro podría tener problemas serios de salud para mi hijo/hija

Totalmente en desacuerdo-------1---

Parcialmente en desacuerdo-----2---

Parcialmente de acuerdo---------3---

Totalmente de acuerdo-----------4---

**Barreras (invertido)**

7. Estoy en la capacidad de almacenar alimentos y materiales lejos de las mascotas y los roedores

Totalmente en desacuerdo-------1---

Parcialmente en desacuerdo-----2---

Parcialmente de acuerdo---------3---

Totalmente de acuerdo-----------4---

8. Estoy en la capacidad de mantener mis animales (gallinas, cabras, cerdos) en corrales fuera de la casa.

Totalmente en desacuerdo-------1---

Parcialmente en desacuerdo-----2---

Parcialmente de acuerdo---------3---

Totalmente de acuerdo-----------4---

**Beneficios**

9. Proteger a los miembros de mi familia de las picaduras del chinchorro es importante para mí.

Totalmente en desacuerdo-------1---

Parcialmente en desacuerdo-----2---

Parcialmente de acuerdo---------3---

Totalmente de acuerdo-----------4---

10. Tener la casa lo más moderna posible es importante para mí.

Totalmente en desacuerdo-------1---

Parcialmente en desacuerdo-----2---

Parcialmente de acuerdo---------3---

Totalmente de acuerdo-----------4---

Totalmente de acuerdo-----------4---

**Creencia de la eficacia**

11.Puedo identificar chinchorros fácilmente

Totalmente en desacuerdo-------1---

Parcialmente en desacuerdo-----2---

Parcialmente de acuerdo---------3---

Totalmente de acuerdo-----------4---

12. Puedo atrapar chinchorros fácilmente

Totalmente en desacuerdo-------1---

Parcialmente en desacuerdo-----2---

Parcialmente de acuerdo---------3---

Totalmente de acuerdo-----------4---

13. Puedo matar chinchorros fácilmente

Totalmente en desacuerdo-------1---

Parcialmente en desacuerdo-----2---

Parcialmente de acuerdo---------3---

Totalmente de acuerdo-----------4---

**Intenciones**

14. Evitaré que los chinchorros entren a mi casa

Totalmente en desacuerdo-------1---

Parcialmente en desacuerdo-----2---

Parcialmente de acuerdo---------3---

Totalmente de acuerdo-----------4---

15. En los próximos seis meses, tengo la intención de evitar que los chinchorros entren en mi casa

Totalmente en desacuerdo-------1---

Parcialmente en desacuerdo-----2---

Parcialmente de acuerdo---------3---

Totalmente de acuerdo-----------4---
